# Ribosome Structure, Function, and Early Evolution

**DOI:** 10.3390/ijms20010040

**Published:** 2018-12-21

**Authors:** Kristopher Opron, Zachary F. Burton

**Affiliations:** 1 Bioinformatics Core, University of Michigan, Ann Arbor, MI 48109-0674, USA; kopron@gmail.com; 2 Department of Biochemistry and Molecular Biology, 603 Wilson Rd., Michigan State University, MI 48824-1319, USA

**Keywords:** EF-G, EF-Tu, coevolution, genetic code, IF2, ribosome, translation elongation, translation initiation, translocation

## Abstract

Ribosomes are among the largest and most dynamic molecular motors. The structure and dynamics of translation initiation and elongation are reviewed. Three ribosome motions have been identified for initiation and translocation. A swivel motion between the head/beak and the body of the 30S subunit was observed. A tilting dynamic of the head/beak versus the body of the 30S subunit was detected using simulations. A reversible ratcheting motion was seen between the 30S and the 50S subunits that slide relative to one another. The 30S–50S intersubunit contacts regulate translocation. IF2, EF-Tu, and EF-G are homologous G-protein GTPases that cycle on and off the same site on the ribosome. The ribosome, aminoacyl-tRNA synthetase (aaRS) enzymes, transfer ribonucleic acid (tRNA), and messenger ribonucleic acid (mRNA) form the core of information processing in cells and are coevolved. Surprisingly, class I and class II aaRS enzymes, with distinct and incompatible folds, are homologs. Divergence of class I and class II aaRS enzymes and coevolution of the genetic code are described by analysis of ancient archaeal species.

## 1. Introduction

We offer a general and conceptual review of translation in prokaryotic systems. We concentrate on prokaryotes in order to correlate ribosome structure and function with the earliest evolution of translation systems. Ribosomes are considered in bacterial systems, using the *Thermus thermophilus* ribosome as an example, and numbering throughout is for *T. thermophilus*. Discussion of transfer ribonucleic acid (tRNA), genetic code, and aminoacyl-tRNA synthetase (aaRS) evolution relies on ancient archaeal species because these functions are the most primitive in archaea. Although some factors have been substituted in evolution, translation mechanisms are similar in archaeal, bacterial, and eukaryotic systems. In our discussion, we stress the homologous binding and functions of IF2, EF-Tu, and EF-G G-protein GTPases in translation initiation and elongation. As part of an ancient pre-ribosome, the 30S subunit and the decoding center appear to be older than an associated 50S subunit, and the 50S subunit may have been recruited from a captured, formerly mobile peptidyl transferase center (PTC). The messenger ribonucleic acid (mRNA) and tRNAs lie across the neck of the 30S subunit between the body and the head/beak. The head/beak reversibly swivels and/or tilts relative to the body to function as a translocation ratchet. The 30S and 50S subunits reversibly rotate relative to one another to form an additional ratchet for elongation and initiation. Interactions between the 30S and 50S subunits and events at the A-site latch regulate translocation. A channel forms between the 30S and the 50S subunits, allowing tRNAs to align, advance, and pass through without major obstructions. We describe translation initiation and elongation using images taken from recent structures. Characteristics of tRNAs that make a relatively stiff and efficient translation adapter are discussed. Translation systems appear to have evolved around tRNA. The referencing in this review is not exhaustive, and we apologize to anyone whose original work we may have overlooked. Excellent and more detailed reviews are available for initiation [1], elongation [2,3,4,5,6,7,8,9], and translocation [10,11], and we refer the reader to these for a more comprehensive referencing of original literature. 

## 2. Initiation of Translation

### 2.1. Homologous GTPases in Initiation and Elongation of Translation

Through the initiation and elongation phases of translation, homologous G-protein GTPases IF2, EF-Tu and EF-G play a central role [1,10,12,13,14]. A homology search shows that these three G-proteins are among the four closest homologs in *T. thermophilus*, along with elongation factor 4, an apparent EF-G mimic [15]. These GTPases interact homologously with the ribosome, particularly with the GTPase-associated complex (GAC) and the sarcin-ricin loop (SRL) of the 50S subunit. An overlay of relevant structures shows the G-proteins and their homologous residues binding to the same site on the ribosome (see below). 

### 2.2. Mechanism of Initiation

Initially, the 30S and 50S ribosomal subunits are dissociated. The initiation complex can form on the 30S subunit before recruitment of the 50S subunit [1], possibly indicating that the 30S ribosomal subunit and its decoding center may, in some sense, be older evolutionarily than the 50S subunit. To bring the 30S and 50S subunits together involves: (1) assembly of initiation factors IF1, IF2, and IF3; (2) the binding of the initiator fMet-tRNA^fMet^ (acylated with *N*-formyl-methionine); and/or (3) the binding of the mRNA on the 30S subunit [16,17]. There may be three primary translocation ratchets for the ribosome. Within the 30S subunit, the head/beak swivels relative to the body. Also, the 30S head/beak can tilt relative to the 30S body. The 50S subunit rotates reversibly relative to the 30S subunit during initiation and elongation. Dynamic motions of the ribosome are important for positioning fMet-tRNA^fMet^ for initiation and also for the stepwise translocation of the mRNA and tRNAs during elongation.

**Figure 1:**
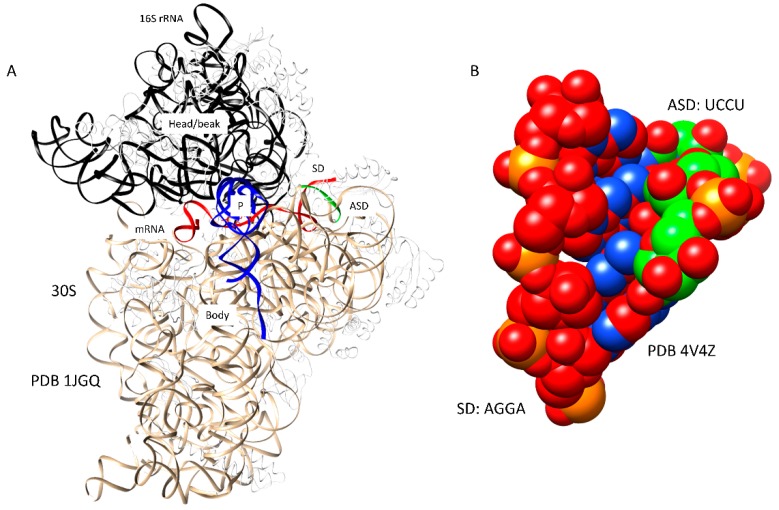
The Shine-Dalgarno-anti-Shine-Dalgarno (SD-ASD) contact in translation initiation on the 30S ribosomal subunit; (**A**) messenger ribonucleic acid (mRNA, red) with an SD sequence binds the ASD (green) near the 3′-end of the 16S rRNA (PDB 1JGQ). The 16S rRNA is beige (body) and black (head/beak). mRNA lies across the neck. tRNA^fMet^ (blue) binds in the P-site. Ribosomal proteins are white; (**B**) detail of the SD-ASD [red-green (carbons)] interaction (PDB 4V4Z).

**Figure 2:**
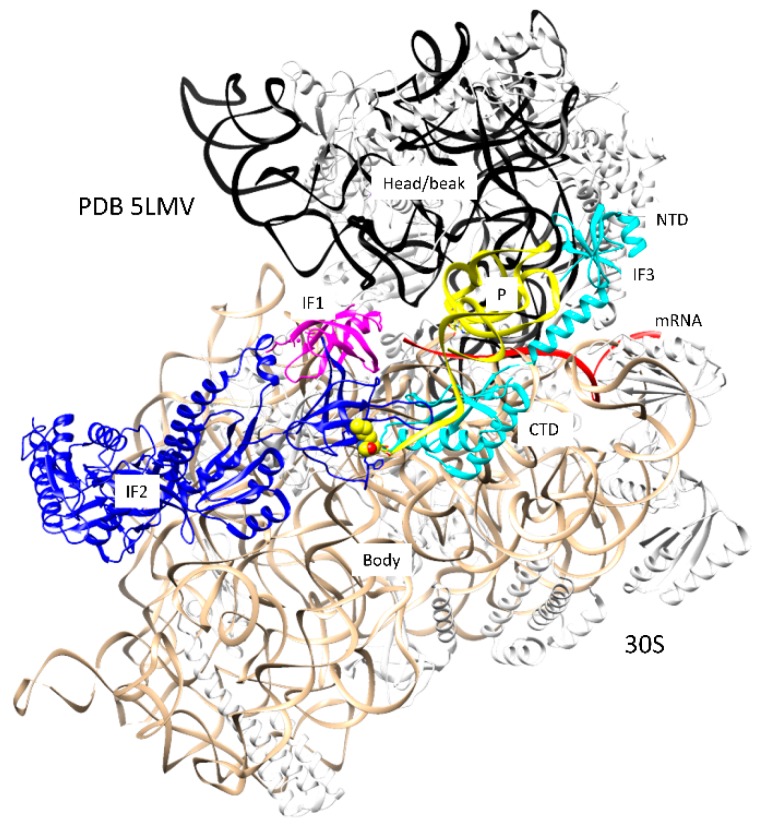
Formation of a pre-initiation complex on the 30S ribosomal subunit (PDB 5LMV). IF1 (magenta), IF2 (blue), and IF3 (cyan) are present. The fMet-tRNA^fMet^ (yellow) is bound in the P-site. Other colors are as in Figure 1. The fMet of the P-site fMet-tRNA^fMet^ bound to IF2 is in the space-filling representation.

Multiple pathways can lead to assembly of the initiation complex (Figure 1 and Figure 2). A Shine-Dalgarno sequence on mRNA can bind to the ribosome independent of initiation factors and fMet-tRNA^fMet^ [1,18]. The images in Figure 1 indicate the formation of the SD-ASD (Shine-Dalgarno-anti-Shine-Dalgarno) interaction [18]. An SD on mRNA (e.g., AGGA) pairs with the ASD near the 3′ end of the 16S rRNA (1537-UCCU) [19], helping to position the mRNA AUG initiation codon at the ribosome P-site. The SD-ASD interaction persists through the assembly of the 50S subunit and early elongation. mRNAs that lack a Shine-Dalgarno sequence can be translated, but these mRNAs probably require previous assembly of IF1, IF2, IF3, and fMet-tRNA^fMet^. The fMet-tRNA^fMet^ is held in the P-site of the 30S subunit by the initiation factors (Figure 2) [16,17]. IF1 appears to block the A-site, preventing assembly of an initiation complex before the 50S subunit recruitment. IF1 helps to bind, orient, and position IF2 and IF3. The NTD (*N*-terminal domain) and CTD (C-terminal domain) of IF3 span the ribosome P-site to position and hold the fMet-tRNA^fMet^ in place. The NTD of IF-3 blocks the E-site to prevent E-site tRNA binding. The CTD of IF3 is highly dynamic in its interactions with the 30S subunit and appears to have a role in positioning the P-site fMet-tRNA^fMet^. Assembly of intermediate complexes stimulates the swiveling of the 30S subunit head/beak [17]. Assembly with the 50S subunit induces relative rotation of the 50S and 30S subunits, helping to accommodate the P-site fMet-tRNA^fMet^ for initiation [20]. Presumably, assembly with the 50S subunit and the release of initiation factors set up conditions to recruit the A-site aa-tRNA, which will enter bound to the IF2 homolog EF-Tu, after the IF2 release, and rotate into the fully accommodated A-site to initiate peptide bond synthesis.

## 3. Elongation of Translation

### 3.1. Molecular Motor

Considered as a molecular motor, the ribosome appears to be driven by a complex thermal ratchet translocation mechanism with an overall step length of ~14 angstroms [1 codon (3 nt) on an extended mRNA] [21]. Because the mRNA is read in the 5′→3′ direction, the mRNA threads through the ribosome in the 3′→5′ direction during stepwise translocation. The ribosome A- (aminoacyl), P- (peptidyl), and E- (exit) tRNA sites are located at the interface (neck) between the 30S subunit body and the head/beak. The mRNA threads through the same crevice (across the neck) in the 16S rRNA as if threaded halfway around a spool. Thus, the mRNA is a neckless (half a choke chain) and the A-, P-, and E-site tRNAs are baubles attached to the chain [22]. There is an ~18° swiveling motion of the 16S rRNA head and beak versus the body (rotation and/or displacement of the neck) that drives ribosome bound tRNAs into hybrid states and then slides back versus the mRNA that stays in an advanced position. Acting as pawls, tRNA 3′-CCA and codon-anticodon [mRNA-ASL (anticodon stem-loop)] interactions appear to maintain the directionality and phase of the ratchet. The exiting peptide chain may also function as a pawl for translocation [23]. Another aspect of the ratchet is a reversible ~7° rotation of the 30S versus the 50S ribosomal subunit, which appears to assist mRNA and tRNA displacement [22,23]. Observed from above the 50S subunit looking toward the 30S subunit beneath, forward translocation is a counterclockwise rotation of the 30S subunit relative to the 50S subunit. The ribosome is a weak molecular machine that generates only ~13±2 pN (pico Newton) of force [21]. 

**Figure 3:**
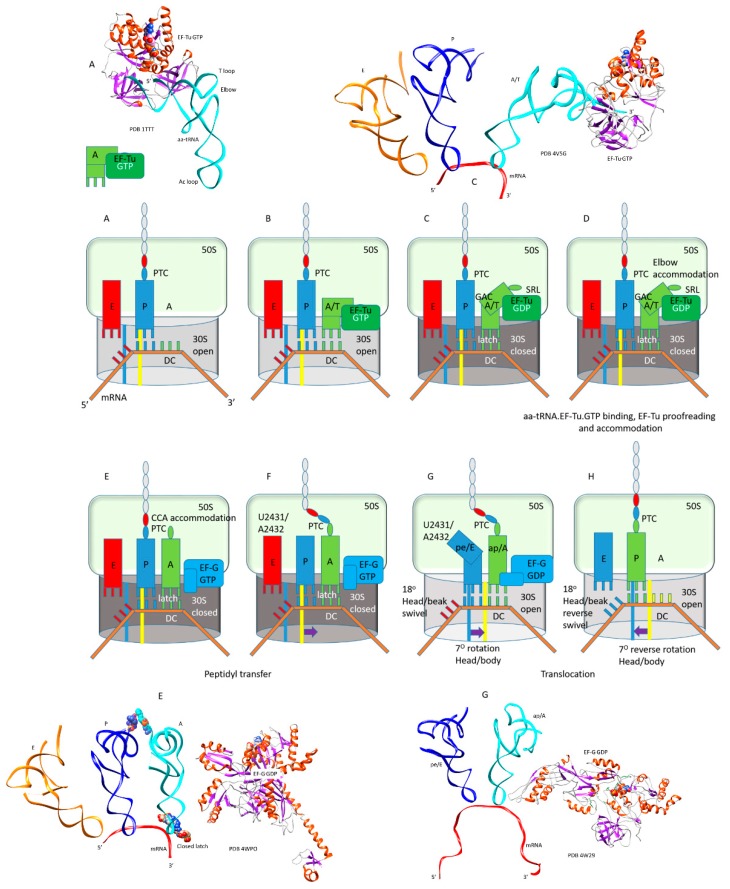
Stages of the translation elongation cycle; (**A**,**B**) binding of the aa-tRNA·EF-Tu·GTP ternary complex to the A/T site; (**C**) conformational closing of the 30S subunit and forming the codon-anticodon A-site latch; (**D**) elbow accommodation of aa-tRNA; (**E**) full CCA accommodation of aa-tRNA to the A/A-site, release of EF-Tu·GDP, entry of EF-G·GTP; (**F**) peptidyl transfer; (**G**) EF-G·GTP→GDP and onset of translocation, opening of the codon-anticodon latch, formation of hybrid tRNA states pe/E and ap/A or ap/ap; (**H**) full forward and reverse translocation. Some intermediate x-ray or cryo-electron microscopy structures are shown.

**Figure 4:**
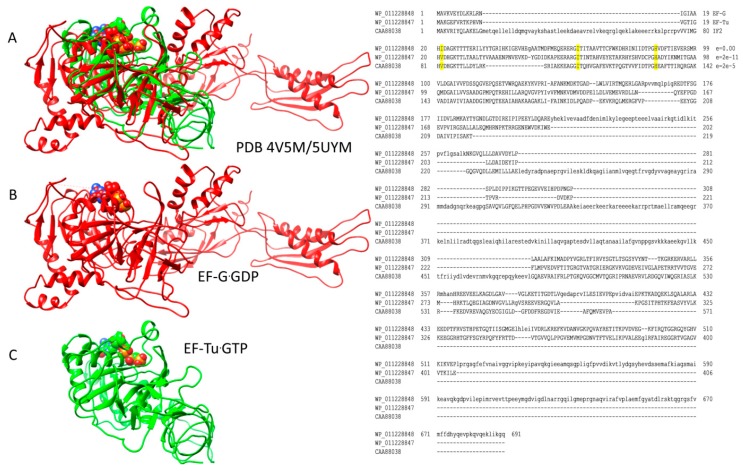
EF-Tu and EF-G are homologs that occupy the same site on the ribosome; (**A**) overlay of ribosome-EF-Tu·GTP (green; PDB 5UYM) and ribosome-EF-G·GDP (red; PDB 4V5M). Overlays were done for ribosomal protein S2. Ribosomes are omitted from the images for simplicity; (**B**) EF-G·GDP structure (red); (**C**) EF-Tu·GTP structure (green). At the right, an alignment of *Thermus thermophilus* EF-G, EF-Tu, and IF2 is shown. Conserved residues (i.e., EF-Tu, Val21, Ile61, and His86) involved in stimulating GTP hydrolysis are indicated. In the alignment, e-values are versus *Escherichia coli* EF-G.

Translation elongation is described in a schematic (Figure 3). As noted above, IF2, EF-Tu, and EF-G are G-protein GTPase homologs that occupy the same site on the ribosome, thus only one of these initiation or elongation factors can be present in a particular intermediate. Therefore, during each peptide bond addition cycle, EF-Tu and EF-G must cycle on and off the ribosome. In Figure 4, ribosome structures were overlaid for ribosomal protein S2, and EF-Tu·GTP and EF-G·GDP from each ribosome structure precisely align for homologous residues and the GTP/GDP-binding site. As noted above, IF2, EF-Tu, and EF-G comprise a set of the closest homologs in *T. thermophilus*. In Figure 3, intermediates A and B describe the aa-tRNA·EF-Tu·GTP ternary complex entry to the ribosome. Intermediates C and D describe the EF-Tu proofreading of the codon-anticodon interaction and elbow accommodation (see below). Intermediates C, D, and E describe the tRNA A-site CCA accommodation, which is entry of the A-site aa-tRNA into proximity to the P-site peptidyl-tRNA for peptidyl transfer. Intermediates E and F describe peptidyl transfer in the presence of EF-G·GTP. Intermediates G and H describe translocation in the presence of EF-G leading to EF-G release. Hybrid tRNA states are indicated as the (codon-anticodon position)/(the 3′-CCA position), i.e., pe/E with the mRNA-ASL in a pe hybrid state (p→e) and the 3′-CCA in the full E-state (Table 1). In Figure 3, some intermediates are highlighted with images from cryo-electron microscopy and X-ray crystallography. The rate of each amino acid addition is ~7 s^−1^, and accommodation appears to be the rate-limiting step [24,25,26]. Defining contacts for the tRNA anticodon loops and 3′-CCA ends are recorded in Table 1 and in the text.

### 3.2. tRNA as a Relatively Stiff Adapter

The tRNA folds into an L-shape in solution. The bend of the L is the elbow, at which the D loop and T loop interact to form a relatively stiff joint. One end of the L is the anticodon (Ac) loop and the other is the 3′-CCA end to which the amino acid is attached. The anticodon loop is a compact 7-mer loop with a U-turn between the 2nd and 3rd loop bases [27]. The 7-mer U-turn loop is essential to present a 3 nt anticodon. A 6- or 8-mer loop, for instance, would not support a U-turn or a 3 nt genetic code. The relative stiffness of tRNA at the elbow and anticodon makes tRNA an adequate adapter for translation. 

Distortions of tRNAs, however, occur during the aa-tRNA accommodation [28] and translocation [29]. tRNA navigates a channel through the ribosome that is approximately the dimensions of a tRNA, but minor obstructions are encountered. Interactions of aa-tRNA with EF-Tu induce tRNA bending [28]. Interaction with the 50S helix89 (h89) during elbow accommodation causes aa-tRNA deformation [30]. During translocation, the tRNA contacts the A-site finger [31] and other micro-pawls [32]. The 3′-CCA-aa end of tRNA is single-stranded and flexible. During CCA accommodation, the 3′-CCA-aa end must navigate to the appropriate position in the PTC [30,33]. 

During codon-anticodon latching and A/A-site tRNA accommodation and translocation, tRNA mostly maintains its characteristic L-shape and rotates to assume new positions. tRNA binds the ribosome as an aa-tRNA·EF-Tu·GTP ternary complex with its aminoacylated end (3′-CCA-aa) bound to EF-Tu [the A/T state (aminoacyl/Ternary Complex)] [34] and rotates into the A/A-site (fully accommodated) after releasing from EF-Tu·GDP [30,33,35,36]. The 3′-CCA-aa end of the A/A-site tRNA is flexible and must penetrate the PTC for peptide synthesis within a dehydrated environment [36,37]. With minor exceptions, therefore, tRNA L-shapes rotate into position rather than undergoing large conformational distortions. Recognition of tRNA rotations between hybrid states is sensed by the ribosome to stimulate sequential events associated with the amino acid addition cycle, some examples being: (1) in transit, tRNAs encounter micro-pawls; (2) the A-site latch opens and closes; (3) the 30S-50S subunit contacts change during rotation; and (4) EF-Tu and EF-G GTPases enter and exit regulating the timing of GTPase activities. 

### 3.3. tRNA Entry

EF-Tu·GTP binds aa-tRNA to form the aa-tRNA·EF-Tu·GTP ternary complex, and this is the form in which aa-tRNA enters the ribosome [34]. EF-Tu·GTP binding sequesters the 3′-CCA-aa end of the incoming aa-tRNA, thus the aa-tRNA cannot enter the fully accommodated A/A-site directly without first binding accurately within the ternary complex to mRNA and then releasing EF-Tu·GDP. EF-Tu and EF-G are activated for GTPase activity by the ribosome, acting as a GTPase-activating factor (GAF). In particular, contact of aa-tRNA·EF-Tu·GTP with the 23S sarcin-ricin loop [SRL; h95: A2660, G2661, A2662, G2663] and the GTPase-associated complex (GAC; ribosomal protein L11, h42, h43, h44, and A1067) of the 50S ribosomal subunit stimulates the EF-Tu GTPase activity, leading to aa-tRNA and EF-Tu·GDP dissociation and elbow accommodation. From simulations, elbow accommodation appears to be a reversible step that dissociates aa-tRNA from EF-Tu·GDP, but EF-Tu·GDP does not appear to dissociate from the ribosome until full CCA-aa accommodation. The aa-tRNA elbow (the bend of the L) interacts with the 50S GAC, which acts as an allosteric effector to stimulate EF-Tu·GTP→GDP [5,38,39,40,41,42]. With initial ternary complex contact to the ribosome, no EF-Tu·GTP contacts are made to the 50S subunit (complex 1, Table 1). Closing of the A-site anticodon stem loop (ASL) latch and hybrid A/T-site aa-tRNA rotation brings aa-tRNA·EF-Tu·GTP into contact with the 50S GAC and the SRL, and these contacts stimulate GTP hydrolysis. Dissociation of aa-tRNA from EF-Tu·GDP during elbow accommodation allows subsequent full CCA-aa accommodation and seating of the aa-tRNA for peptidyl transfer. EF-G·GTP stimulates peptide bond formation, indicating replacement of EF-Tu·GDP with EF-G·GTP before peptide bond synthesis. EF-G·GTP→GDP stimulates translocation [10,22]. 

### 3.4. Forming the Accurate Codon-Anticodon Latch and Closing the 30S Subunit Conformation

**Figure 5:**
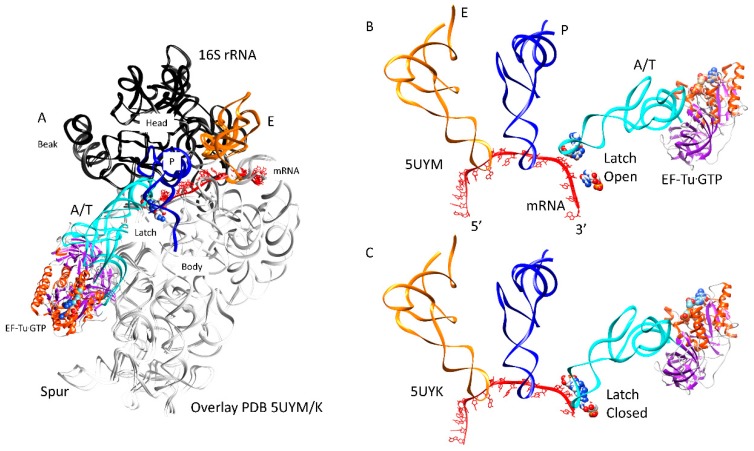
Closing of the codon-anticodon latch closes the 30S ribosomal subunit; (**A**) overlay of open and closed latch structures. The head and beak are black (16S: 930–1380). The body is white. tRNA sites locate to the cleft between the head/beak and the body; (**B**) the latch is open (complex 1); (**C**) the latch is closed (complex 3). This transition occurs in three stages (Table 1).

The accuracy of translation requires the tight closing of the A-site codon-anticodon latch to confirm base pairing (Figure 5). An overlay of open and closed latch structures is shown in Figure 5A. The overlay image is shown to emphasize that mRNA threads along the 16S rRNA neck (h28) between the head/beak and body. An open conformation of the latch is shown in Figure 5B [43], and a closed 30S conformation is shown in Figure 5C [44]. The latch is comprised of the A-site codon-anticodon helix and the 16S rRNA nucleotides G530 (G530 loop), A1492 and A1493 (h44), and the 23S rRNA nucleotide A1913 (h69). The ribosomal protein S12 interacts with the closed latch. The latch closure involves G530~A1492 H-bonding and G530 H-bonding to tRNA wobble and central anticodon position ribose rings [43]. Sealing the latch closes the 30S subunit by bringing the 16S rRNA G530 loop next to the 16S rRNA h44 (A1492, A1493). The closing of the A-site latch is communicated to the 50S subunit through interactions of the 30S h44 (A1492, A1493) and the 50S h69 (A1913), h71, and h62. 

By contrast, a near-cognate tRNA generally fails to maintain a closed conformation of the latch and the 30S subunit, releasing and replacing the aa-tRNA·EF-Tu·GTP/GDP ternary complex before inaccurate aa-tRNA CCA accommodation and aa misincorporation can occur. A near-cognate tRNA would be one with a G~U wobble pair in the 1st or 2nd codon position, which correspond to the 2nd or 3rd anticodon position (reading 5′→3′). The codon-anticodon latch, therefore, is tight enough to accurately select Watson-Crick pairs from wobble pairs in the 1st and 2nd codon positions (the 2nd and 3rd anticodon positions). From x-ray crystallography studies, it appears that near cognate G~U pairs within a tightened latch are forced into Watson-Crick geometry, which requires keto → enol tautomerization of either G or U and is not energetically favorable nor kinetically stable, which leads to near-cognate aa-tRNA release [44]. By contrast, wobble contacts are generally allowed in the wobble position, which is the 3rd codon position (the 1st anticodon position). Because wobble base pairs are tolerated at the wobble position of the codon-anticodon, the maximum complexity of the genetic code in tRNA is 2 × 4 × 4 = 32 anticodons versus 4 × 4 × 4 = 64 codons in mRNA (see below) [45,46]. Generally, therefore, tRNA anticodon geometry and readout limited the potential size of the genetic code, because for the most part—with an exception of tRNA^Ile^ (UAU), which is very rarely used in prokaryotes versus tRNA^Met^ (CAU)—only pyrimidine-purine discrimination is possible at the wobble position. 

**Figure 6:**
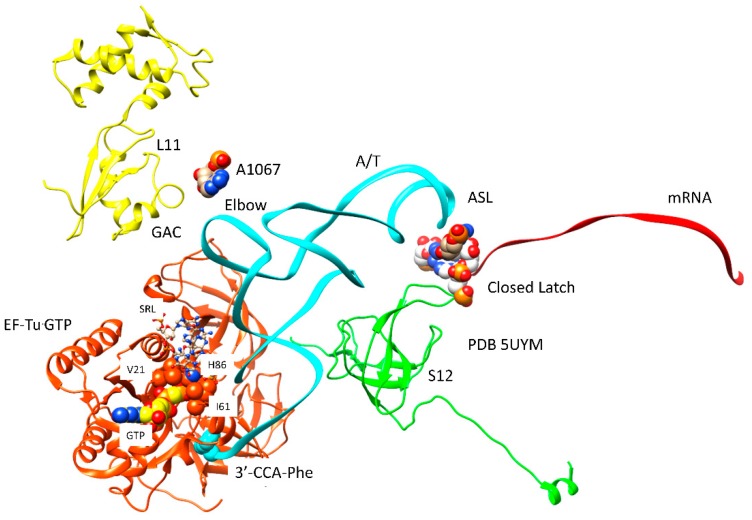
50S GTPase-associated complex (GAC) and SRL (ball and stick representation) binding to aa-tRNA·EF-Tu·GTP activates His86 to stimulate GTPase activity. Numbering of EF-Tu residues is as in Figure 4.

The mechanism of EF-Tu GTPase activation by the 50S ribosomal subunit is shown in Figure 6 [43]. The 23S rRNA SRL has a central role because contact with the SRL opens a hydrophobic gate on EF-Tu that is formed by interaction of Ile61 (Switch 1) and Val21 (P loop; G1 box). Opening the hydrophobic gate allows His86 (Switch 2) to approach the GTP γ-phosphate and activate a water molecule to engage in hydrolysis. Because GTP hydrolysis requires the 50S SRL interaction with aa-tRNA·EF-Tu·GTP, which is dependent on the prior 30S subunit closing and sealing the codon-anticodon latch, EF-Tu regulates the accuracy of codon-anticodon binding and latching and is an initial check on translational accuracy. The A-site codon-anticodon latch remains closed through: (1) EF-Tu GTP hydrolysis and dissociation; (2) accommodation of the aa-tRNA into the A/A-site; (3) EF-G·GTP binding; and (4) formation of the peptide bond. The A-site latch, therefore, can be considered to be a powerful pawl in regulating A-site to P-site translocation, because the latch must open to allow this progression. The regulatory and catalytic residues in EF-Tu are conserved in its homologs and replacements EF-G and IF2 (Figure 4), as are contacts on the ribosome to the SRL and GAC.

### 3.5. Accommodation

**Figure 7:**
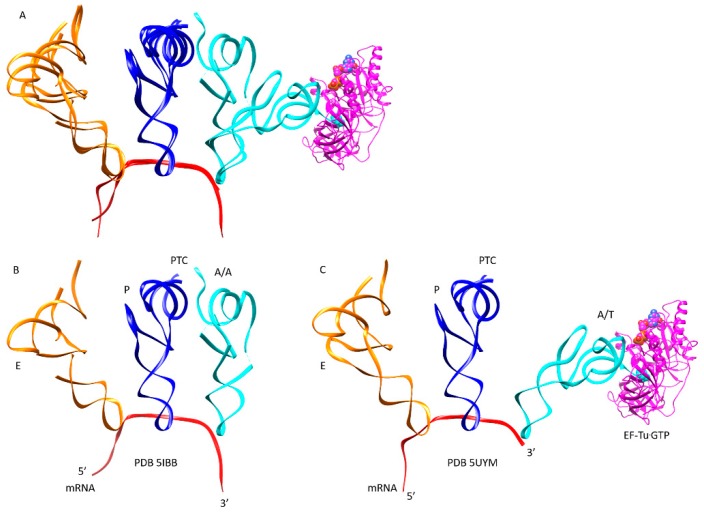
Accommodation of aa-tRNA from the A/T-site to the A/A-site. This is a multi-step process that includes elbow accommodation and CCA accommodation (see Figure 3; Table 1); (**A**) overlay of PDB 5IBB (A/A state) and 5UYM (A/T state); (**B**) the fully accommodated A/A state poised for peptidyl bond formation; (**C**) the A/T state before elbow accommodation and CCA accommodation. EF-Tu·GTP is colored magenta.

Accommodation requires a large rotation of the A-site aa-tRNA from the A/T-site to the A/A-site (~100 angstrom transition of the incoming amino acid) (Figure 7). Figure 7A shows an overlay of A/T and A/A structures. Figure 7B shows the fully accommodated A/A structure. Figure 7C shows the A/T structure before the release of EF-Tu from aa-tRNA, elbow accommodation, and CCA accommodation.

Accommodation is described in two major steps: (1) elbow accommodation; and (2) CCA accommodation [28,30,33]. It appears that EF-Tu·GDP remains associated with the ribosome through aa-tRNA elbow accommodation, which is a major step in proofreading the accuracy of the anticodon-codon attachment. Elbow accommodation indicates that the A-site tRNA elbow (the bend of the L) is positioned proximal (within ~30 angstroms) to the P-site tRNA elbow. To obtain this position, the aa-tRNA elbow must interact and slide against the h89 of the 50S ribosomal subunit, an interaction that is stimulated by the presence of EF-Tu·GDP, although aa-tRNA must dissociate from EF-Tu·GDP to access the elbow accommodated position. The second step is CCA accommodation, which involves the complete seating of the 3′-CCA-aa within the peptidyl transferase center (PTC). For full A/A-site aa-tRNA accommodation, EF-Tu·GDP dissociates from the ribosome, allowing the entry of EF-G·GTP, which stimulates peptidyl transfer. 

Once EF-Tu dissociates, the free 3′-CCA-aa end of the A-site tRNA is single-stranded and flexible and must penetrate the PTC [30,35,36,37]. It appears that this transition may occur via multiple routes. In the A/A-site, the C75 of the aa-tRNA forms a restraining Watson-Crick base pair with 23S G2553 (h92, A-loop, PTC). The C75:G2553 contact can be considered a defining aspect of the A-site tRNA in the PTC. The P-site tRNA is similarly restrained at its 3′-CCA-peptide end by G:C Watson-Crick base pairs (G2252:C74 and G2251:C75). Accommodation is viewed as the primary proofreading step in translation because cognate aa-tRNA survives the long transition but near-cognate and non-cognate aa-tRNAs, which are less stable at the ASL, dissociate [2,30,44]. Because EF-Tu appears to associate with the ribosome through both the A-site aa-tRNA latching step and the elbow accommodation steps that are most important for proofreading the ASL-mRNA contact, EF-Tu is a major fidelity factor for translation. 

### 3.6. Peptide Bond Formation

**Figure 8:**
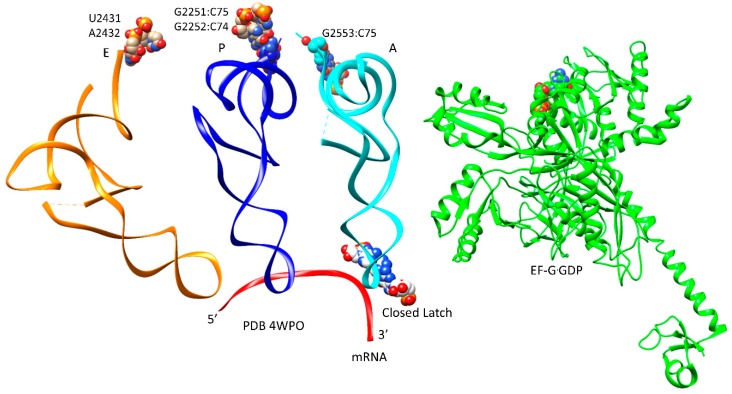
EF-G·GDP (green) in a compact conformation in a pre-translocation or catalytic state. The compact conformation may be more indicative of an EF-G·GTP catalytic structure. Note the induced kink or bend in the mRNA at the latch that helps position P-site and A-site tRNAs in sufficient proximity for peptidyl transfer.

The codon-anticodon A-site latch remains closed through peptidyl bond formation, but opens to enable translocation. CCA accommodation results in a kink in the mRNA between the P-site and A-site tRNAs in order to position the tRNA 3′-ends for peptidyl transfer (Figure 8). After bond formation, the latch relaxes to allow translocation of the A-site tRNA to the P-site. The P-site 3′-CCA-peptidyl-tRNA is restrained by PTC bases (P-loop) G2251 that pairs peptidyl-tRNA C75, and G2252 that pairs C74 (Watson-Crick pairs). A2451, C2452, and U2585 also make defining P-site peptidyl-tRNA contacts. These P-site 3′-CCA-peptide contacts appear to break only after peptidyl transfer, EF-G GTPase activity, and the onset of translocation (Figure 8). Onset of translocation transfers the 3′-CCA end of the deacylated P-site tRNA into the E-site (U2431, A2432) and its anticodon loop into a pe hybrid state [23,47]. 

Bringing and finally locking the 3′-CCA-aa end of the A/A-site tRNA close to the P-site tRNA peptide in a dehydrated environment with appropriate molecular positioning and crowding is sufficient to support peptide bond synthesis. The peptide chain is transferred from the P-site tRNA to the A-site tRNA, where the peptide chain mostly remains stationed. In the presence of EF-G·GTP/GDP, reversible excursions between A-site and P-site contacts are observed for peptidyl-tRNA, described as A/A, ap/A, and ap/ap hybrid states [47,48]. In the ap/A←→ap/ap hybrid state, G2553 of the A loop (h92) either forms a Watson-Crick base pair to peptidyl-tRNA C75, which is typical of an A-site contact, or G2251:C75 and G2252:C74 P-loop (h80) Watson-Crick pairs are observed, which are typical of a P-site contact. This dynamic conversion appears to continue until late in translocation [49]. After full translocation, the peptidyl-tRNA resides in the P-site. It may be that a longer peptide chain, which is missing in most structures, could act as a stronger pawl, driving forward translocation of peptidyl-tRNA from the A-site to the P-site. 

The PTC is considered to be a molecular crowding and dehydration chamber generally lacking any particular ribozyme activity [50]. Although the PTC has been described as a ribozyme, it is not a good one [49]. It has been reasonably suggested that a generalist bond formation function of the PTC helps to support peptidyl transfer using 20 encoded amino acid substrates. Peptide bond formation is stimulated by EF-G·GTP, thus EF-G·GTP enters the complex and contacts the closed latch before peptide bond formation occurs but after the homolog EF-Tu·GDP release from the shared binding site. For cognate aa-tRNA incorporation, accommodation appears to be the overall rate-limiting and proofreading step for translation [51,52].

The ribosome PTC reaction is described as a two pathway mechanism [53,54]. One pathway is the proton shuttle mechanism in which a zwitterionic transition state is generated after A-site aa-tRNA attack. The proton shuttle mechanism appears to be the major route for protein synthesis, at least with the substrates utilized in these studies (i.e., A-site Phe-tRNA^Phe^ and P-site fMet-tRNA^fMet^). A high kinetic solvent isotope effect (hydrogen/deuterium exchange) for protein synthesis indicates proton transfers from water, supporting the proton shuttle pathway. Potentially, some amino acid substrates may prefer the alternate reaction pathway, which is general base catalyzed. The pH dependence of the protein synthesis mechanism so far observed, however, is not generally consistent with heavy use of the base-catalyzed path. In contrast to peptide synthesis, translation termination mechanisms utilize the base-catalyzed path, leading to P-site peptidyl-tRNA hydrolysis and peptide chain release mediated by a protein translation release factor [54,55,56]. Because translation termination appears to use the base-catalyzed path, and because the base-catalyzed path does not appear to be energetically inaccessible, the base-catalyzed pathway may also be used for some peptide synthesis reactions. 

### 3.7. Translocation

**Figure 9:**
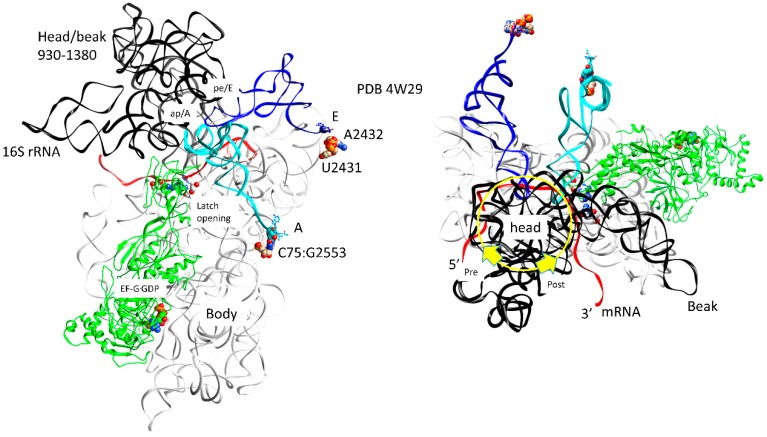
EF-G GTPase activity and translocation. The tRNA pe/E and ap/A (or ap/ap) hybrid states are observed. The 16S rRNA nucleotides 930–1380 are black (head/beak domain). mRNA (red) occupies the channel between the 16S rRNA body and head/beak (the neck). The codon-anticodon latch is not fully closed and the ap/A tRNA anticodon stem loop (ASL) has disengaged from the latch and has begun to translocate toward the P-site. For the ap/A-site tRNA, the 3′-CCA, where a peptide chain would be attached, makes a typical CCA A-site contact (C75:G2553). The pe/E tRNA 3′-CCA makes a typical E-site contact (U2431, A2432). EF-G·GDP is in an extended conformation supporting forward translocation and acting as a pawl to prevent reverse translocation.

**Figure 10:**
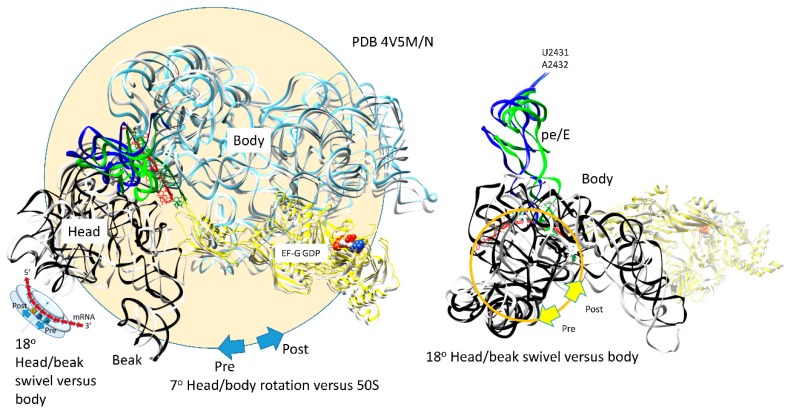
Two thermal rotary motions of the 30S subunit support forward translocation. The 30S subunit reversibly rotates ~7° versus the 50S subunit. The mRNA [red (post translocated) and green (pre-translocated)] threads between the 16S rRNA head/beak domain and the body (the neck), ~18° swiveling of the head/beak domain versus the body drives the mRNA forward and the mRNA:tRNA codon:anticodon attachments into hybrid states. For the pre-translocated state, mRNA and pe/E tRNA are green. 16S rRNA (pre) is white. For the post-translocated state, EF-G·GDP (extended conformation), mRNA, and pe/E site tRNA are yellow, red, and blue. The 16S rRNA (post) is light blue (body) or black (16S head/beak; 930–1380). In the rightmost image, the 16S rRNA body is not shown to simplify the image.

The ribosome has been discussed as a relatively weak molecular motor with a complex thermal translocation ratchet (Figure 9 and Figure 10) [21]. The ratchet has three primary modes: (1) the 30S head/beak swivel; (2) the 30S head tilt; and (3) the reversible rotation of the 30S and the 50S subunits relative to one another. Other micro-motions are also identified, such as: (1) the opening and closing of the L1 stalk; (2) the movements of the L11 stalk to regulate GTPases; and (3) the 30S subunit h44 dynamics. In Figure 9, translocation motions are indicated for a single intermediate. In Figure 10, two intermediate structures (designated pre and post) are overlaid [22]. The ratchet is biased forward by a P-site tRNA rotating its 3′-CCA end into the E site, EF-G GTPase activity and, after translocation, reverse ratcheting and swiveling and EF-G·GDP release. A-site, P-site, and E-site tRNAs are located at the interface of the 16S rRNA head and body (Figure 9). The mRNA also threads through the cleft separating the head/beak from the body of the 16S rRNA (across the neck; h28). Swiveling of the 16S head/beak by ~18° appears to advance the mRNA and move tRNAs into hybrid states. For instance, swiveling of the head/beak dissociates the E-site tRNA and moves the P-site tRNA into a pe/E hybrid conformation that makes defining E-site contacts at its 3′-CCA end (23S U2431, A2432) but makes a pe hybrid transition at its ASL. Forward swiveling of the head occludes the empty A-site so the reverse swiveling head/beak motion becomes part of the tRNA binding, latching, and accommodation processes for formation of the next peptide bond.

The 30S and the 50S subunits of the ribosome are somewhat loosely attached. Subunit association is stabilized by mRNA binding and Mg^2+^. The somewhat loose binding allows restrained sliding of the subunits relative to one another. Ratcheting of the 30S subunit relative to the 50S subunit by ~7° helps to drive forward translocation [22]. Before reverse ratcheting and swiveling, the entry of tRNAs that are not in an EF-Tu·GTP complex is inhibited. Movement of the P-site tRNA to the E-site and the entry of aa-tRNA·EF-Tu·GTP support a natural forward directionality and flow to translation. From structures, EF-G·GTP appears to hold the A-site mRNA-ASL codon-anticodon latch for peptidyl transfer. Therefore, EF-G·GTP→GDP might release the A-site latch to support A/A→ap/A←→ap/ap translocation [47,48]. EF-G·GDP likely dissociates from the ribosome after the reverse 30S subunit head/body ratcheting versus the 50S subunit and the reverse 16S rRNA head/beak swiveling relative to the 16S body. Tilting of the 30S head/beak versus the 30S body also occurs in translocation [57]. From simulations, the order of events in translocation appears to be: (1) ratcheting of the 50S subunit relative to the 30S subunit; (2) swiveling of the 30S head/beak relative to the 30S body; and (3) the 30S head tilt.

### 3.8. EF-G·GTP/GDP in Translocation

EF-G·GTP/GDP undergoes multiple conformational distortions during binding, peptidyl transfer, GTPase, and translocation [3,10,29,58,59]. PDB 4WPO is characterized as a pre-translocation state of the ribosome, but the conformation is very close to that expected of a catalytic state (Figure 8) [60]. Most interestingly, EF-G·GDP is covalently locked in a compact conformation, which is the expected form for EF-G·GTP before GTP hydrolysis and at the time of peptidyl transfer from the P-site to the A-site. In PDB 4WPO, the latch is closed, and the mRNA is kinked between the A-site and P-site, as expected for a catalytic intermediate. Subsequent translocation complexes have; (1) a more elongated conformation of EF-G·GDP; (2) an opening latch; and (3) the A-site tRNA ASL-mRNA contact has moved forward into an ap/A or an ap/ap hybrid position (Figure 9 and Figure 10) [47,48]. Translocating ribosomes have been captured in a number of intermediate states that likely represent EF-G·GTP [29,48] and EF-G·GDP [59] complexes.

### 3.9. 30S-50S Intersubunit Bridges in Translocation

Because the 30S and the 50S subunits rotate relative to one another in translocation, intersubunit contacts regulate dynamics [61,62]. In the 30S subunit, h44 (A-site latch; A1492, A1493) is a long helix that makes multiple contacts to the 50S subunit, including to h69 (B2a; bridge 2a), h71 (B3), and h62 (B6). B2a 50S h69 is part of the A-site latch, including latch residue A1913. B3 is very close to the center of the ratchet for intersubunit rotation. B6 is very important to maintaining the 30S-50S association. The 30S h44 connects the A-site ASL latch (16S G530, A1492, A1493, 23S A1913) with the 50S subunit (h69). Mutations that disable bridges to 16S h44 are lethal. The 30S S13 protein interacts with the 50S h38 (A-site finger) (B1a). This contact affects the dynamics of the 30S head (i.e., swivel and/or tilt). The 30S S15 protein contacts the 50S h34 (B4). The 30S h23 contacts the 50S h68 (B7a), and the 30S h14 contacts the 50S L14/L19 (B8). Mutations that break B1a, B4, B7a, and B8 accelerate forward and reverse translocation, indicating that these are pawls that help to maintain the mRNA coding and the 30S-50S binding registers. 

### 3.10. Ratchet Pawls

We posit that A-site, P-site, and E-site codon-anticodon (mRNA-ASL), 3′-CCA tRNA attachments, and the exiting peptide chain form the primary pawls that help maintain the ribosome translocation phase. The E-site does not bind acylated tRNA, so tRNA cannot slip from the P-site to the E-site without the transfer of the peptide chain to the A-site. As might have been expected, mutation of the E-site near the 3′-CCA contact causes frameshifting errors [63]. This is anticipated if the E-site evolved (in part) to limit backwards mRNA slippage and to maintain the translocation phase. The exiting peptide chain may also act as a pawl to help enforce the A-site to P-site and the P-site to E-site tRNA transitions. Because only translocation intermediates with hybrid pe/E and E/E tRNAs have been observed, available data do not appear to address the precise mechanism of EF-G·GDP release or to describe the full 14 angstrom translocation step. Often, translocation by molecular machines that is driven by thermal ratchets involves a partial power stroke (i.e., EF-G·GTP→GDP) followed by forward sliding and the establishment of pawls to inhibit backtracking. Maintaining 2-3 tRNAs on mRNA during translation helps to hold the translocation phase. Therefore, the E-site tRNA helps to maintain the register. The peptide chain and the A-site latch appear to help hold the mRNA. Traversing or relaxing pawls helps move the mRNA stepwise through the ribosome. Based on this analysis, at least 5-7 pawls must be considered in addition to any micro-pawls that may influence transitions. Primary pawls in translation correspond to tRNA codon-anticodon (mRNA-ASL) attachments (2 or 3), tRNA 3′-CCA attachments (2 or 3), and the exiting peptide chain (1). The EF-G·GTP→GDP power stroke and conformational extensions of EF-G have been considered to establish pawls that maintain forward translocation. 

The A-site finger (23S 879-898; h38) that contacts the 30S S13 protein might be considered a micro-pawl to maintain the register for translocation. Transitioning from the A/A-site to the ap/ap-site, tRNAs contact the A-site finger [31]. Type I tRNAs make weaker contact than type II tRNAs (with expanded V loops). Deletion of the A-site finger affects translocation rates and may cause frameshift errors. Another micro-pawl might be the PE loop (16S G1338-U1341). Interaction of the pe/E tRNA ASL with the PE loop and ribosomal protein S13 appears to induce the 30S head tilting [57]. The 16S rRNA C1397 and A1503, which can intercalate with mRNA bases, have also been considered to be micro-pawls to resist reverse translocation [29]. 

### 3.11. Kink-Turns and Micro-Motions

In concert with the larger ratcheting, tilting, and swiveling motions of the ribosome are smaller movements. The ribosome is described as a flexible, semi-stable, and dynamic molecular motor [23]. The 50S and the 30S subunits reversibly slide relative to one another in a reversible ratcheting motion, and the 16S rRNA head/beak swivels relative to the 16S body around the neck [22]. Kink-turns are small loops that cause instabilities in helices, allowing for movements of an arm that may contribute to or resist translocation. Several of these kink-turns have been identified that may be important in regulating GTPase activity and translocation. The GAC that stimulates GTPase activity for IF2, EF-Tu, and EF-G is mounted on a kink-turn (Kt-42) located in 23S rRNA h42. The GAC is comprised of h42, h43, h44, and ribosomal protein L11. A-site to P-site translocation of the decoding center appears to involve an interaction between the 16S rRNA h44 (body) and h28 (neck). This action is associated with: (1) EF-G·GTP→GDP; (2) the opening of the 30S subunit; (3) the opening of the G530~A1492 latch; and (4) the movement of the A-site tRNA codon-anticodon from an A/A-state to a reversible ap/A←→ap/ap hybrid state. This has been observed as an ~8 angstrom movement of the decoding center [23,48]. A kink-turn (Kt-38) in the 23S rRNA h38 is found near a 50S to the 30S subunit contact (affecting the A-site finger interaction with protein S13). 

## 4. Evolution of Translation

### 4.1. tRNA Evolution

**Figure 11:**
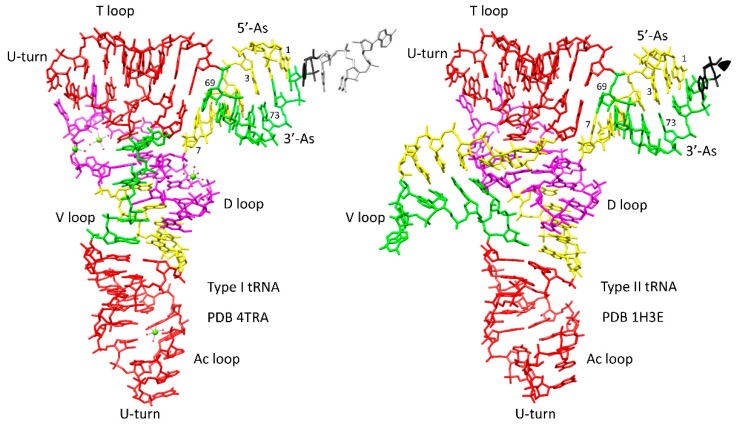
tRNA evolution. Homologous regions have the same color. Ac and T loop stem-loop-stems (17 nt) are red (initially ~CCGGGUUCAAAACCCGG). 5′-As (initially GCGGCGG) and homologous regions are yellow. 3′-As (initially CCGCCGC) and homologous regions are green. The D loop microhelix UAGCC repeat region (originally 17 nt) is magenta. Green spheres are Mg^2+^ ions.

Translation systems evolved around tRNA. Internal tRNA homologies and tRNA evolution are described in Figure 11 for type I and type II tRNAs (type II tRNAs have expanded V loops) [27,64]. The 17 nt Ac (anticodon) loop and T loop stem-loop-stems are homologous (initially close to 5′-CCGGGUU/CAAAACCCGG; sequence ambiguity is only in the 7 nt loop, not in the 5 nt stems; / indicates the position of the U-turn). The position of the U-turn is the same (between loop positions 2 and 3) in the Ac and T loops. The D loop is derived from a 17 nt UAGCC repeat (initially 5′-UAGCCUAGCCUAGCCUA). The last 5 nt of the D loop region (the yellow segment just 5′ of the Ac stem-loop-stem in Figure 11) is homologous to 5′-acceptor stems (As; 5′-As positions 3-7; initially 5′-GGCGG). The 5′-As evolved from a GCG repeat (initially 5′-GCGGCGG). The type I tRNA V loop (5 nt) is homologous to a 3′-As (3′-As nt 69-73 using our revised numbering for tRNAs; initially 5′-CCGCC). The 3′-As evolved from a CGC repeat (initially 5′-CCGCCGC). The type II tRNA V loop (initially 14 nt) is homologous to a 3′-As (7 nt) ligated to a 5′-As (7 nt) (initially 5′-CCGCCGCGCGGCGG).

Type I tRNA evolved by the ligation of three 31 nt minihelices followed by two symmetrical 9 nt deletions within ligated 3′- and 5′-acceptor stems [27,64,65]. Type II tRNA evolved by the ligation of three 31 nt minihelices followed by one 9 nt deletion within the 5′ ligated 3′- and 5′-acceptor stems [64]. Type II tRNA is a proposed intermediate in processing to type I tRNA, and, thus, the same model explains both tRNA subtype variants. A minihelix is a 17 nt microhelix flanked by 5′ and 3′ 7 nt acceptor stems [27,64]. Other models for tRNA evolution have been advanced, but no model based on the ligation of two minihelices [66,67,68,69] can be correct for the many reasons that have previously been explained [27,64]. For instance, in a two minihelix model, the Ac and T loops cannot be homologs, and, therefore, only a three minihelix model can describe tRNA evolution. The three minihelix model is strongly supported using statistical tests [27,64]. 

### 4.2. tRNA as Core Evolutionary Intellectual Property

**Figure 12:**
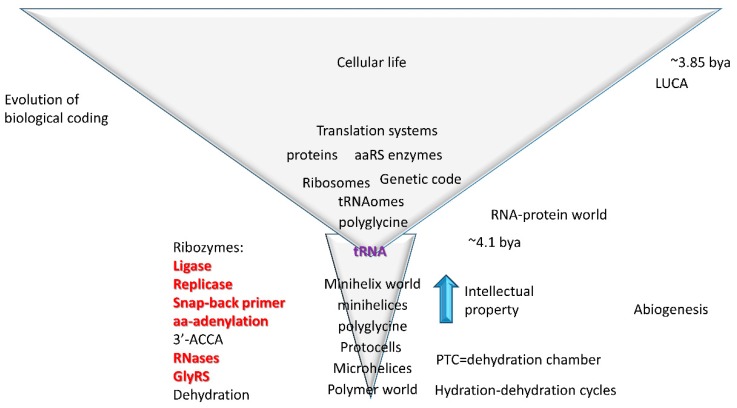
A model for the evolution of abiogenesis, the RNA-protein world, and cellular life. RNA and ribozyme functions that have been generated in vitro are indicated in red. The central advance in evolution of life on earth and biological coding is tRNA. This figure was modified from [64].

As explained above, tRNA evolved from repeats (GCG, CGC, and UAGCC repeats; 5′-As, 3′-As, and D loop) and inverted repeats (~CCGGGUUCAAAACCCGG; Ac loop and T loop stem-loop-stems) [27,64]. In Figure 12, a model for abiogenic evolution of tRNA from repeating polymers and inverted repeats is shown. Only a handful of ribozymes are necessary to generate tRNA from minihelix, microhelix, and repeating polymer precursors. Existing tRNAs radiated from these ordered sequences [27,64]. According to the model, tRNA comprises the central biological intellectual property necessary to coevolve rRNA, mRNA, the genetic code, aminoacyl-tRNA synthetase (aaRS) enzymes, and biological coding. Hydration-dehydration cycles are sufficient to generate many biopolymers [70,71], supporting the idea of the PTC as a dehydration chamber [50]. 

### 4.3. Aminoacyl-tRNA Synthetase Evolution

Aminoacyl-tRNA synthetases (aaRS; i.e., GlyRS) charge tRNAs with amino acids [72]. These enzymes are of two folding classes, designated class I and class II, with structural subclasses A-E (i.e., GlyRS-IIA or IleRS-IA). Class I aaRS enzymes have an active site of parallel β-sheets, described as a “Rossmannoid” fold. Evolutionarily, however, there is no detectable homology comparing class I aaRS enzymes and classical Rossmann fold (β–α)_8_ proteins. By contrast to class I aaRS, class II aaRS enzymes have an active site of antiparallel β-sheets. Rodin and Ohno posited that, despite their incompatible folds, class I and class II aaRS enzymes were somehow related, possibly by antisense transcription-translation [73,74,75,76,77,78]. Class I and class II aaRS enzymes, however, have been shown to be likely homologs [45]. Our laboratory has obtained local alignments of IleRS-IA (i.e., *Methanobacterium bryantii*) and GlyRS-IIA (i.e., *Methanobacterium congolense*) enzymes with e-values as low as 5 × 10^−11^, strongly indicating homology.

**Figure 13:**
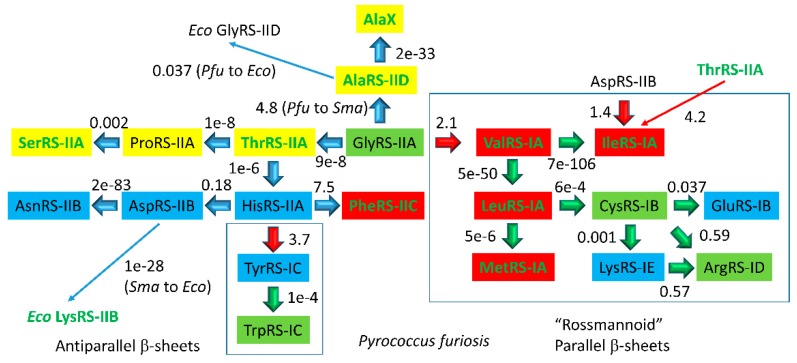
Aminoacyl-tRNA synthetases (aaRS) evolution (mostly) in *Pyrococcus furiosis (Pfu)*, an ancient archaea. Columns of the genetic code are indicated by shading: column 1 (red); column 2 (yellow); column 3 (blue); and column 4 (green). Class I and class II aaRS enzymes are indicated with their structural subclasses (A–E). aaRS enzymes with editing active sites are in green type. Boxes are placed around class I aaRS enzymes. *Sma: Staphylothermus marinus; Eco: Escherichia coli*. AlaX is an editing function missing a synthetic AlaRS active site. This figure is modified from [46].

**Figure 14:**
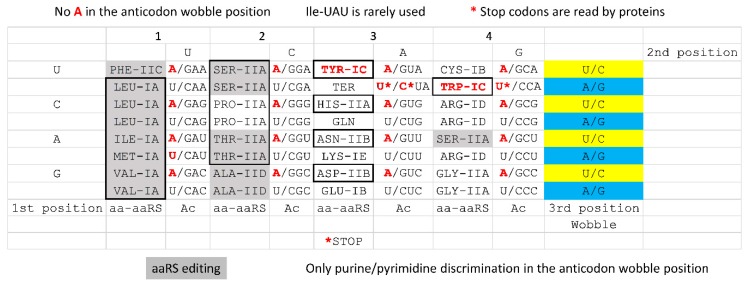
In archaea, the genetic code is effectively half as complex in tRNA compared to mRNA. The genetic code is shown as a codon-anticodon (Ac) table. The structural classes of aaRS enzymes are indicated (i.e., GlyRS-IIA is indicated as GLY-IIA). Grey shading indicates aaRS editing. Red bases are not utilized in the tRNA anticodon wobble position. Boxes indicate co-evolution of amino acids and aaRS enzymes in columns. TyrRS-IC and TrpRS-IC (boxed) are related across rows. Significantly, only pyrimidine/purine discrimination is achieved in the wobble position in archaea because of anticodon wobble ambiguity. Ancient archaea tend to have ~44 tRNAs, but tRNA wobble U and C anticodons are effectively synonymous.

In Figure 13 and Figure 14, archaeal aaRS enzymes are compared. In Figure 13, the comparison is mostly for *Pyrococcus furiosis*, an ancient species that is very similar to LUCA (the last universal common cellular ancestor). Because of the proximity of *P. furiosis* to LUCA, this comparison gives an accessible and qualitative view of early aaRS evolution and radiation. Figure 13 indicates: (1) aaRS editing; (2) aaRS structural subclasses; (3) closest apparent relatives (by e-value); and (4) genetic code columns (see also Figure 14). We make the following points from Figure 13: (1) related aaRS enzymes tend to align in genetic code columns; (2) class I and class II aaRS enzymes appear to be homologs; and (3) aaRS editing is mostly confined to columns 1 and 2 of the genetic code and to hydrophobic (column 1) and neutral (column 2) amino acids, for which accurate aaRS discrimination in the active site may be problematic. Evolution of the genetic code appears to be via tRNA charging errors, and the code goes to universality and closure because of translational fidelity mechanisms that inhibit continued code innovation by suppressing tRNA charging errors [46]. 

The smaller the e-value, the more closely related are the enzymes. From column 1 of the genetic code (Figure 13 and Figure 14), ValRS-IA, IleRS-IA, MetRS-IA, and LeuRS-IA are closely related class IA enzymes that attach hydrophobic amino acids (Val, Ile, Met, Leu) and that edit improper amino acid attachments. Editing by aaRS enzymes involves moving a mischarged aa-tRNA into a proofreading active site and hydrolysis of the inappropriate amino acid attachment. Very clearly, in column 1, hydrophobic amino acids, aaRS-IA enzymes, aaRS editing, and the genetic code structure are co-evolved. From column 2, ThrRS-IIA, ProRS-IIA, and SerRS-IIA are related class IIA enzymes. ThrRS-IIA and SerRS-IIA edit amino acid attachments. Thr and Ser are closely related neutral amino acids with the capacity for one hydrogen bond to the amino acid side chain, limiting the specificity of ThrRS and SerRS discrimination of substrates. ThrRS-IIA appears to be closely related to GlyRS-IIA, which may indicate that a GlyRS once occupied column 2 of the code (see below). In column 3, HisRS-IIA, AspRS-IIB, and AsnRS-IIB appear related, indicating additional code structure. The closely related TyrRS-IC and TrpRS-IC that charge tRNAs with aromatic amino acids locate to the top row of the genetic code, possibly indicating late evolution of the code along rows rather than within largely occupied columns. Phe, Tyr, and Trp are thought to be some of the last amino acids added to the code. We conclude that amino acids, aaRS enzymes, tRNAs, and the genetic code structure are coevolved, as expected. These strong patterns and structures in the genetic code are most apparent when represented as a codon-anticodon table in ancient archaea, such as *P. furiosis*, which is similar to LUCA. 

### 4.4. rRNA Evolution

rRNA is posited to have evolved after and around tRNA [79,80]. The ribosome may have initially evolved as a scaffold for mRNA (pre-16S rRNA) utilizing a mobile PTC, which was a dehydration and molecular crowding chamber to enable peptide bond formation [50,65]. With the evolution of the 23S rRNA, the PTC became immobile and the translocation ratchet (~7° rotation) could be evolved between the two ribosome subunits. The tRNA passage channel, which is formed in the 50S ribosomal subunit, probably evolved around tRNA to mold the channel shape and size to allow transit of tRNAs. In evolution, major blocks to tRNA passage would have been strongly negatively selected. Minor tRNA-interacting features of the ribosome (i.e., the L1 stalk, the A-site finger, and the P-site finger) may have evolved as pawls to help maintain the mRNA reading frame. The 16S rRNA swivel between the head/beak and the body, therefore, may have been the most primitive translocation mechanism for a standalone pre-16S rRNA scaffold before the recruitment, evolution, and attachment of the 50S subunit. The long 30S h44 connects to the 50S subunits h69, h71, and h62, linking the A-site ASL latch to the 50S subunit and the translocation mechanism. 

### 4.5. Evolution of the Genetic Code

A representation of the genetic code is shown in Figure 14. The code is displayed as a codon-anticodon table with emphasis on the reduced effective size of the genetic code in tRNA relative to mRNA. Representation of the anticodon is essential because the genetic code evolved around tRNAs [45,46]. For instance, adenine is disallowed in the tRNA anticodon wobble position [45,46]. In archaea and bacteria, moreover, tRNA^Ile^ (UAU) is rarely used. Furthermore, single base discrimination of A, G, C, and U at the anticodon wobble position is difficult. Effectively, only purine versus pyrimidine discrimination is achieved at the wobble position for archaeal tRNAs. At the base of code evolution, therefore, the genetic code capacity in tRNA shrinks to 32 distinct anticodons at most [45,46]. The near universal standard genetic code encodes 20 amino acids + stops. The complexity of the standard code is limited due to the maintenance of 4-codon sectors, which are protected from subdivision by amino acid identity and aaRS editing—two issues of aaRS charging accuracy and translational fidelity. Hydrophobic and neutral amino acids with limited capacity for hydrogen bonding tend to locate to 4-codon sectors (columns 1 and 2) rather than splitting into 2-codon sectors (column 3). Stop codons are recognized by proteins, not tRNAs, so Trp should not be considered to reside in a 1-codon sector. In archaea, tRNA^Ile^ (UAU) is rarely used, so tRNA^Ile^ (UAU) and tRNA^Met^ (CAU) should not be considered 1-codon sectors at the base of code evolution. We prefer the classic structure and representation of the genetic code to circular code diagrams because code structure is most apparent using a codon-anticodon table (Figure 14). 

The most primitive ribosomes and tRNAs are posited to have synthesized polyglycine [45,46,81,82] using any RNA as an mRNA template. Polyglycine is currently a cross-linking component of bacterial cell walls [45,46] and may have had a similar role in stabilizing protocells before LUCA. From such beginnings, the genetic code, which appears to be the crowning achievement of abiogenesis (Figure 12), could be evolved by straightforward Darwinian selection. We posit that Gly was the first encoded amino acid that, at an early stage of evolution, Gly occupied the entire genetic code table [45,46]. Also, SerRS-IIA is split in the code between column 2 and column 4, indicating that, as the code evolved, Ser occupied blocks from which it was subsequently excluded, as we propose for Gly. If the entire genetic code initially encodes glycine, this has two important consequences. First, initially all mRNA sequences encode polyglycine. Second, according to this model, the genetic code, tRNA, and mRNA coevolve via Darwinian selection to a complete and accurate code. 

The oldest rRNA sequences appear to be more tRNA-like than more derived species, indicating that tRNA existed before the evolution of 16S and 23S rRNA. The 16S rRNA appears to be more similar in sequence to tRNA than 23S rRNA and 5S rRNA. From a 1-letter code in which every mRNA encodes polyglycine, the code can progress from a 1-letter→4-letter→8-letter→16-letter→21-letter (20 aa + stops) code, as previously described [45,46]. Evolution of the A-site codon-anticodon latch in the 16S rRNA may correspond to the 8-letter→16-letter code transition [45,46]. The 2nd nt in the codon-anticodon position is most important for translational accuracy. However, the latch was necessary to discriminate the 1st codon position (the 3rd anticodon position). This discrimination was required to generate a code complexity of 4 × 4=16-letters. Because the latch also supports accuracy of pairing at the wobble position, evolution of the latch supported the further evolution from a 16-letter to a 21-letter code, bringing the standard genetic code to closure and universality. For other views, see [83,84]. 

## 5. Conclusions

The ribosome is a large and dynamic thermal ratchet molecular motor at the heart of information processing in cells. The mRNA and bound tRNAs traverse the 16S rRNA neck. Translocation is supported by a reversible swiveling motion of the 16S rRNA head/beak versus the body, by an apparent tilting of the head/beak relative to the body, and by a reversible ratcheting motion between the 50S and the 30S ribosomal subunits. Directionality of translation appears to be due to GTPase elongation factors EF-Tu and EF-G that bind in turn to the same site on the ribosome. EF-Tu is a primary fidelity factor for translation. EF-G mostly functions in peptidyl transfer and translocation. Attachments of tRNAs to mRNA and to the 50S subunit appear to act as pawls to enforce the direction and the mRNA register of translation. The 30S-50S subunit contacts regulate translocation and appear to communicate events at the A-site ASL latch to translocation ratchets. The coevolution of ribosomes, the genetic code, mRNA, and aaRS enzymes appears to have been around the tRNAs that evolved from ordered repeat sequences and stem-loop-stems that include a U-turn within a 7 nt loop (Ac and T loop). 

## Figures and Tables

**Table 1:** Phases of the Translation Elongation Cycle.

Intermediate	Stage	Ternary Complex	EF-G	A Site		P Site		E Site		PDB	Proofreading
(Figure 1)				ASL	CCA/PTC	ASL	CCA/PTC	ASL	CCA		
A		aa-tRNA.EF-Tu.GTP (free)		empty	empty	P site	A2451, C2452, U2585, G2252:C74, G2251:C75, CCA-peptide	E site	E site U2431, A2432	1TTT	
B	Cplx 1	aa-tRNA.EF-Tu.GTP (bound)		A/T, open	empty	P site	A2451, C2452, U2585, G2252:C74, G2251:C75, CCA-peptide	E site	E site U2431, A2432	5UYK	
	Cplx 2	aa-tRNA.EF-Tu.GTP (bound)		A/T, latched	empty	P site	A2451, C2452, U2585, G2252:C74, G2251:C75, CCA-peptide	E site	E site U2431, A2432	5UYL	!!
C	Cplx 3	aa-tRNA.EF-Tu.GTP (bound)		A/T, latched	empty	P site	A2451, C2452, U2585, G2252:C74, G2251:C75, CCA-peptide	E site	E site U2431, A2432	5UYM	!!
D	Elbow	aa-tRNA.EF-Tu.GDP (bound)		EA, latched	empty	P site	A2451, C2452, U2585, G2252:C74, G2251:C75, CCA-peptide	E site	E site U2431, A2432		!!!!
E	CCA		EF-G.GTP (binds)	A, latched	CCA-aa, C75:G2553	P site	A2451, C2452, U2585, G2252:C74, G2251:C75, CCA-peptide	E site	E site U2431, A2432	5IBB, 4WPO	
F			EF-G.GTP	A, latched	CCA-peptide, C75:G2553	P site	A2451, C2452, U2585, G2252:C74, G2251:C75, CCA	E site	E site U2431, A2432		
G	pre		EF-G.GDP	ap, open	(CCA-peptide, C75:G2553)	empty	(ap/A<-->ap/ap tRNA-peptide)	pe	pe/E tRNA: U2431, A2432	4W29, 4V5M	
	pre/post		EF-G.GDP	ap, open	(CCA-peptide, C75:G2553)	empty	(ap/A<-->ap/ap tRNA-peptide)	pe	pe/E tRNA: U2431, A2432		
H	post		EF-G.GDP	ap, open	(CCA-peptide, C75:G2553)	empty	(ap/A<-->ap/ap tRNA-peptide)	pe	pe/E tRNA: U2431, A2432	4V5N, 5OT7	
latch: 30S: S12, 16S: G530~A1492, A1493; 23S: A1913											

Cplx: complex; elbow or EA: elbow accommodation; CCA: CCA accommodation. Steps that are most important for translational accuracy are indicated with exclamation points.

## Abbreviations

MDPI	Multidisciplinary Digital Publishing Institute
DOAJ	Directory of open access journals
aaRS	Aminoacyl-tRNA synthetase (i.e., GlyRS)
A-site	Aminoacyl site
As	Acceptor stems
ASL	Anticodon stem loop
Ac loop	Anticodon loop
EF	Elongation Factor
E-site	Exit site
IF	Initiation Factor
LUCA	Last universal common (cellular) ancestor
P-site	Peptidyl site
PTC	Peptidyl Transferase Center
SRL	Sarcin-ricin loop
T loop	T loop or TΨC loop
V loop	Variable loop
